# Nitrogen availability impacts oilseed rape (*Brassica napus* L.) plant water status and proline production efficiency under water-limited conditions

**DOI:** 10.1007/s00425-012-1636-8

**Published:** 2012-04-08

**Authors:** Benjamin Albert, Françoise Le Cahérec, Marie-Françoise Niogret, Pascal Faes, Jean-Christophe Avice, Laurent Leport, Alain Bouchereau

**Affiliations:** 1 UMR 1349 Institut de Génétique, Environnement et Protection des Plantes, INRA, Agrocampus Ouest, Université de Rennes 1, 35653 Le Rheu cedex, France; 2 UMR 950 Ecophysiologie Végétale, Agronomie et Nutritions NCS, INRA, Université de Caen Basse-Normandie, 14032 Caen, France

**Keywords:** *Brassica napus*, Drought stress, Nitrogen supply, Proline metabolism, Source-sink relationship, Water status

## Abstract

Large amounts of nitrogen (N) fertilizers are used in the production of oilseed rape. However, as low-input methods of crop management are introduced crops will need to withstand temporary N deficiency. In temperate areas, oilseed rape will also be affected by frequent drought periods. Here we evaluated the physiological and metabolic impact of nitrate limitation on the oilseed rape response to water deprivation. Different amounts of N fertilizer were applied to plants at the vegetative stage, which were then deprived of water and rehydrated. Both water and N depletion accelerated leaf senescence and reduced leaf development. N-deprived plants exhibited less pronounced symptoms of wilting during drought, probably because leaves were smaller and stomata were partially closed. Efficiency of proline production, a major stress-induced diversion of nitrogen metabolism, was assessed at different positions along the whole plant axis and related to leaf developmental stage and water status indices. Proline accumulation, preferentially in younger leaves, accounted for 25–85 % of the free amino acid pool. This was mainly due to a better capacity for proline synthesis in fully N-supplied plants whether they were subjected to drought or not, as deduced from the expression patterns of the proline metabolism *BnP5CS* and *BnPDH* genes. Although less proline accumulated in the oldest leaves, a significant amount was transported from senescing to emerging leaves. Moreover, during rehydration proline was readily recycled. Our results therefore suggest that proline plays a significant role in leaf N remobilization and in N use efficiency in oilseed rape.

**Electronic supplementary material:**

The online version of this article (doi:10.1007/s00425-012-1636-8) contains supplementary material, which is available to authorized users.

## Introduction

Winter oilseed rape (*Brassica napus* L.) is a major crop cultivated worldwide mainly for oil for human consumption and renewable fuel. It is very efficient in taking up mineral nitrogen (N) from the soil profile for vegetative growth during the autumn–winter period (Malagoli et al. 2005). Conventional crop management practice requires the use of relatively high amounts of N fertilizers, from 150 to 300 kg of N ha^−1^, to ensure an optimum yield (Rathke et al. 2006), as the nitrogen use efficiency (NUE) of this crop is low (Schjoerring et al. 1995; Dreccer et al. 2000). A number of traits are targets in an effort to significantly increase NUE such as improved rooting and greater root length density at depth (Svecnjak and Rengel 2006), prolonged N uptake mainly after flowering (Teakle et al. 2008), optimized stem N storage and delayed senescence (Malagoli et al. 2005), low seed protein and high seed oil contents (Nyikako 2003) and low leaf N loss (Malagoli et al. 2005). The low NUE of oilseed rape has been significantly associated with the weak N remobilization that occurs in leaves during vegetative development (Dejoux et al. 2000; Malagoli et al. 2005) and is affected by senescence (Aufhammer et al. 1994; Desclos et al. 2009), mainly due to the loss of N-rich leaves (Schjoerring et al. 1995; Dreccer et al. 2000; Rossato et al. 2001).

Pollution by excessive application of fertilizers and nitrate leaching is a source of environmental and economic concern in agriculture, and new goals are being set to reduce N supply while genetically improving the NUE of crops including oilseed rape (Aufhammer et al. 1994; Barlóg and Grzebisz 2004; Masclaux-Daubresse et al. 2008). However, lowering N input while optimizing yield may be counterproductive if it compromises how crop plants respond to environmental challenges, like high temperature and low water availability. In areas with a temperate climate, oilseed rape is often exposed to seasonal drought, and according to global warming forecasts, such climatic vagaries will limit crop productivity in the near future.

In oilseed rape, the negative effects of drought are quite similar to those for N limitation, with loss of grain yield and depreciation of all yield components (Andersen et al. 1996; Champolivier and Merrien 1996; Norouzi et al. 2008). Seed oil content declines after drought (Danesh-Shahraki et al. 2008) and lipid composition changes (Bouchereau et al. 1996). Furthermore, water stress slows leaf development (Qaderi et al. 2006), restricts stomatal conductance (Mogensen et al. 1997) and reduces leaf photosynthetic capacity (Gammelvind et al. 1996; Müller et al. 2010). Loss in productivity may be even more consequential when N input is lower as drought tolerance of oilseed rape may largely depend on N availability. N metabolism in leaves is particularly sensitive to drought stress, which causes a fall in protein and polar lipid content (Ilami and Contour-Ansel 1997; Benhassaine-Kesri et al. 2002) and a rise in amino acid content (Good and Zaplachinski 1994). Oilseed rape subject to water stress is notably able to accumulate very large amounts of free proline (Trotel et al. 1996; Larher et al. 2003; Norouzi et al. 2008).

Proline accumulates as a compatible osmolyte during water stress and may also serve as a readily accessible source of carbon, nitrogen and energy when it is remobilized or oxidized during recovery (Trotel et al. 1996; Szabados and Savouré 2010). Under favourable conditions, proline can also act as a signal molecule that controls the expression of numerous genes and influences plant growth and development, including flowering and seed set (Mattioli et al. 2008; Székely et al. 2008). Proline is synthesized mainly from glutamate (Székely et al. 2008) by Δ^1^-pyrroline-5-carboxylate (P5C) synthetase (P5CS; EC 2.7.2.11) and P5C reductase (P5CR; EC 1.5.1.2) and converted back into glutamate by proline dehydrogenase (PDH; EC 1.5.99.8) and P5C dehydrogenase (P5CDH; 1.5.1.12). In the Brassicaceae *Arabidopsis thaliana*, P5CS and PDH are encoded by two genes and P5CR and P5CDH by single ones (Strizhov et al. 1997; Funck et al. 2010; Servet et al. 2012). High proline concentrations have been measured in the phloem sap of drought-stressed plants, e.g. in *Medicago sativa* (Girousse et al. 1996) while glutamate and glutamine are the most abundant amino acids in the phloem of oilseed rape when it is well watered (Lohaus and Moellers 2000). Thus, proline is closely connected to the glutamate–glutamine metabolism which is of primary importance in the context of N remobilization efficiency and foliar senescence prematurely induced by environmental factors, such as water or N deficiency. Proline can also be derived from ornithine by ornithine δ-aminotransferase (δ-OAT; EC 2.6.1.13) in an alternative pathway.

To investigate how low N input management might affect oilseed rape production, the present study aimed at assessing how this species responds to water stress under different N fertilizer regimes. First, the physiological impact of drought stress on leaf ranks of the plant rosette grown under high or low N regimes was compared by analysing a range of parameters commonly used to screen for drought tolerance. The potential of oilseed rape to recover from water deficit period was then assessed with respect to N availability throughout a rehydration phase. Focusing on N use efficiency and N remobilization performance, proline metabolism regulation at the whole plant level was investigated as (1) a major accumulated metabolite component of drought tolerance possibly affected by the plant N status and (2) an organic N resource which could participate or interfere with the processes of nutrient remobilization at the whole plant level and NUE depending on growth conditions. These working hypotheses were examined through (1) the collection of leaf blades and phloem saps at different positions of the plant and experiments carried out with individual leaf explants and (2) the measurements of proline content and relative expression of *BnP5CS* and *BnPDH* genes.

## Materials and methods

### Plant material and growth conditions

Three-week-old seedlings of oilseed rape *Brassica napus* L. genotype Tenor (seeds were kindly provided by CETIOM, Centre de Grignon, Thiverval-Grignon, France) were individually planted into 2 l pots filled with perlite. All plants were grown in a greenhouse with a thermo/photoperiod of 25 °C/16 h days and 18 °C/8 h nights and natural light supplemented to ensure a minimum photosynthetically active radiation of 200 μmol photons m^−2^ s^−1^ at the top of the leaf canopy.

### Nitrogen treatment, watering and sampling of plants

**Fig. 1:**
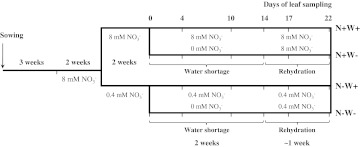
Summary of oilseed rape culture conditions and experimental workflow designed to apply contrasted nitrogen and/or water treatments. N+W+, control plants received 8 mM nitrate and were watered throughout; N−W+, N-deprived plants received 0.4 mM nitrate for 5 weeks and were watered throughout; N+W−, water-stressed plants received 8 mM nitrate but were not watered for 2 weeks from day 0; N−W−, N and water-deprived plants received 0.4 mM nitrate when watered but were not watered for 2 weeks from day 0

For 2 weeks (Fig. 1), plants were regularly watered to full capacity with a total of 8 mM nitrate as nitrogen source in N+ solution [3 mM KNO_3_, 2.5 mM Ca(NO_3_)_2_, 1 mM MgSO_4_, 0.5 mM KH_2_PO_4_, 30 μM H_3_BO_3_, 27 μM FeNa-EDTA, 10 μM MnSO_4_, 1 μM ZnSO_4_, 1 μM Na_2_MoO_4_, 0.5 mM CuSO_4_ and 0.5 μM Co(NO_3_)_2_]. Just after emergence of the seventh leaf, plants were fertilized with high (8 mM nitrate) or low (0.4 mM nitrate) amounts of N, respectively, with N+ solution or N− solution (0.4 mM KNO_3_, 3 mM KCl, 2.5 mM CaCl_2_, 1 mM MgSO_4_, 0.5 mM KH_2_PO_4_ and the same micro-nutrients as those in N+ solution). Irrigation was adjusted to the growth requirements of the two sets of plants, making sure that excess water did not remain in trays underneath pots until just before the application of water shortage. After 2 weeks under contrasting N regimes, half of the plants were deprived of water (W−) by completely stopping irrigation for 2 weeks when plants had wilted. Half of the plants were irrigated as before (W+) until the end of the experiment. Irrigation of water-stressed plants was restarted on day 15 and continued for 8 days. Treatments were referred to as N+W+ (control), N−W+ (low N only), N+W− (water stress only) and N−W− (low N and water stress).

Five plants per treatment were sampled after 2 weeks of contrasted N nutrition (day 0), after 4, 10 and 14 days of water shortage and after 3 and 8 days of rehydration (i.e. at days 0, 4, 10, 14, 17 and 22). This experimental sampling program was conducted for the measurement of leaf water status indices, physiological traits and proline contents in leaves and phloem exudates. Analysis of the *BnP5CS* and *BnPDH* gene expression levels was performed after 10 days of drought (see below). The leaf disc assays were realized from plants submitted to only 10 days of contrasted N regimes and the duration of water deprivation was shortened to 7 days to allow the application of an additional hyper-osmotic stress (see below). For all plants, each leaf rank was identified and numbered based on the date of ontogenetic appearance from the oldest to youngest (Dornbusch et al. 2011).

### Measurement of leaf water status and other physiological parameters

The global impact of N and water treatments on the dynamics of plant growth was monitored by counting the total number of leaves at each sampling date, noting whether they had newly emerged, remained attached the plant, or had fallen. Parameters of water and physiological status were measured on four leaf ranks #6, #9, #12 and #15 that at day 0 of the experiment corresponded to senescent, mature, young and emerging leaves, respectively.

Water content (WC) and relative water content (RWC) were calculated as follows: WC (%) = 100 × (FW − DW)/FW, and RWC (%) = 100 × (FW − DW)/(TW − DW), where FW is the fresh weight, DW the dry weight and TW the turgid weight, i.e. the weight of the fully hydrated leaf (obtained after 4 h of hydrating the leaf in darkness).

Leaf water potential (Ψ_w_) was determined as the petiole xylem pressure potential using the Scholander-type pressure chamber (Model 1000, PMS Instrument Co., Albany, OR, USA). Leaf osmotic potential (Ψ_S_) was estimated by measuring osmolality of crushed leaf juice with a freezing-point osmometer (Model 13DR, Roebling, Berlin, Germany).

Leaf chlorophyll content was calculated as the mean of four independent readings using the non-destructive chlorophyll meter SPAD (SPAD-502 model, Minolta, Tokyo, Japan), which measures leaf transmittance in two wavelength regions (650 and 940 nm) of maximum light absorbance by chlorophyll a and b (Konica Minolta Sensing Inc. 2003). Maximum photosynthetic efficiency (i.e. the maximum quantum yield of PSII, *F*_v_/*F*_m_) was monitored with the Handy PEA portable chlorophyll fluorescence analyser (Hansatech Instruments Ltd, Norfolk, UK). Chlorophyll fluorescence was measured in the flag leaf blades after 10 min of dark adaptation. The measurements were made at a saturating photon flux density (1,500 μmol photon m^−2^ s^−1^) for 1 s by ultra-bright red LEDs optically centred to a peak wavelength of 650 nm.

Stomatal conductance (*g*_s_), expressed in mmol m^−2^ s^−1^, was estimated using the LC*i* compact portable photosynthesis system (ADC BioScientific Ltd, Hertfordshire, UK), which also measures the net photosynthetic rates of leaves (over a 6.25 cm² area) under the ambient CO_2_ concentration (approximately 400 ppm) at 25 °C.

### Collection of phloem exudates

Plants exclusively dedicated to the collection of phloem sap exudates were sampled after 2 weeks of water shortage under both N regimes. Plants were conditioned for 24 h in a growth chamber under a 16 h light/8 h dark cycle with temperature and relative humidity at 23/20 °C and 70/90 %, respectively. Leaf petioles were cut from one plant for each of the four treatments and quickly rinsed with 20 mM EDTA pH 7.0 to prevent callus formation so phloem contents could exude freely. Petioles were cut again and immersed in 5 ml of EDTA and placed for 60 min in darkness at 20 °C and 70 % relative humidity. After this absorption period, petioles were rinsed and phloem sap was allowed to exude into 5 ml of ultra-pure water for 18 h in light at 23 °C and 90 % relative humidity. Exudates were then frozen in liquid nitrogen, lyophilized and stored at −20 °C for amino acid analysis.

### Determination of proline and total free amino acid (TFAA) contents

Proline and free amino acid contents of phloem exudates and leaves (excluding the petiole and the main midribs) were measured. Sampled leaf tissues were immediately frozen in liquid nitrogen, lyophilized and ground into a fine powder stored at −80 °C. Individual free amino acid contents were determined with the method described by Renault et al. (2010). Amino acids in leaves were first extracted from 10 mg of dry powder with a mixture of methanol–chloroform–water containing a known concentration of DL-3-aminobutyric acid (BABA) as an internal standard. Amino acids were then derivatized with a Waters kit (Waters Corporation, Milford, MA, USA) for analysis by UPLC^®^–DAD (Waters Corporation). Individual amino acids were identified by co-chromatography with pure synthetic compounds (Sigma-Aldrich, St. Louis, MO, USA) and quantified with respect to the BABA signal and individual external calibration curves. Proline and TFAA contents were expressed in μmol g^−1^ DW.

### Leaf disc assays

The capacity of individual leaves to produce or degrade proline was estimated by measuring the proline content of explants (10 mm diameter) taken from leaf ranks corresponding to senescent, mature, young and emerging leaves of N+W+, N−W+, N+W− and N−W− plants 1 week into the water shortage period. Proline content was assayed by the modified colorimetry method with ninhydrin as described by Magné and Larher (1992).

The capacity for proline synthesis was estimated by inducing hyper-osmotic stress at −2.5 MPa. Leaf explants were incubated for 16 h under continuous light (200 μmol photons m^−2^ s^−1^) at 25 °C in a reference medium containing 5 mM Hepes, 1.5 mM CaCl_2_ and 10 mM KCl, pH 6.0 with the addition of PEG 6000 (Larher et al. 1993). Experiments were also performed on leaf discs enriched with glutamine (an efficient substrate for proline synthesis after conversion to glutamate) by a 6-h pre-incubation with 40 mM glutamine in the reference medium.

To determine the capacity for proline degradation, leaf discs were enriched with proline in a 6-h pre-incubation with 40 mM proline in the reference medium, followed by 4 h in the reference hypo-osmotic medium (at −0.04 MPa).

### RNA extraction

RNA was extracted from leaf lamina of plants submitted to 10 days of water deprivation to enable to observe differential levels of gene expression. The RNA isolation protocol based on phenol extraction and lithium chloride precipitation was applied as described by Verwoerd et al. (1989). The quantity and quality of RNA samples were assessed by spectrophotometry with a NanoDrop ND 1000.

### Quantitative reverse transcription-polymerase chain reaction (qRT-PCR) analysis of *P5CS* and *PDH* gene expression

Total RNA (2 μg) treated with RNase-free DNase I (Fermentas, Thermo Fisher Scientific Inc., Waltham, MA, USA) was reverse transcribed with Moloney murine leukaemia virus reverse transcriptase (M-MLV RTase) (Q-BIOgene, MP Biomedicals, Illkirch, France) following the manufacturer’s recommendations. qRT-PCR was performed on diluted first-strand cDNA using the LightCycler 480 SYBR Green I Master kit (Roche Applied Science, Mannheim, Germany). Specific *Brassica napus* primers based on sequences described by Xue et al. (2009) were designed to amplify the *P5CS1* gene (5′-CGATTTGGACTTGGTGCTGA-3′ and 5′-GCCCATCCTCTCCTAGTC-3′) and *P5CS2* gene (5′-CCATTATCTTCCTCCTCTCAC-3′ and 5′-AACAACTGCTGTCCCAACC-3′). For *PDH* cDNA amplification, primer sequences were defined based on an alignment of the full-length *PDH* cDNA sequences from *B. napus*, *B. oleracea* and *B. rapa* (Genbank accessions EU375567, GU568241 and EU186335), enriched with 131 EST selected in a Blast search of Genbank (http://www.ncbi.nlm.nih.gov/Genbank/) for similarity with the *Arabidopsis thaliana* *AtPDH1* sequence *At3g30775*. None of these cDNA or EST sequences showed significant similarity to the *AtPDH2* gene *At5g38710*. The primer pair 5′-GCAGCHCACGTAAGGATC-3′ and 5′-CBGGTTGGAGGATTGTGTC-3′ was designed to specifically amplify all *PDH1* sequences. After 5 min at 95 °C, real-time qRT-PCR was performed by 50 cycles including 15 s at 95 °C and 40 s at 60 °C. Products of 219, 200 and 94 bp were amplified from *BnP5CS1*, *BnP5CS2* and *BnPDH1* cDNAs, respectively, as verified by sequencing (Beckman Coulter Genomics, Essex, UK). Two technical repeats per biological replicate were analysed. PCR efficiencies (*E*) were estimated from calibration curves generated from serial dilutions of a blend of all cDNAs and ranged from 71.8 to 99.3 %. For each gene, the expression level in each sample was referred to the control sample showing the highest expression (lowest crossing point value) according to geNorm’s procedure (http://medgen.ugent.be/genorm/). The relative expression levels of the target genes were then normalized against reference genes *B. napus* *PP2A*, *UBC21* and *PTB* homologous to the Arabidopsis *At1g13320*, *At5g25760* and *At3g01150* genes, respectively (Czechowski et al. 2005). A single, more accurate and reliable normalization factor (NF) was used for the three internal control genes, corresponding to their geometric mean calculated by geNorm (Vandesompele et al. 2002). The final normalized and relative gene expression levels were therefore calculated as follows: *E*_T_^Δ*C*pT(*C*−*S*)^/NF. *E*_T_: efficiency of target amplification, where *C*_pT_ is the cycle number at the target detection threshold (crossing point) for the sample *S* and control *C*.

### Statistical analysis

All calculations were performed with Statistica software (version 7.1, StatSoft Inc., Tulsa, OK, USA, http://www.statsoft.com). The normal distribution of the data was studied with the Shapiro–Wilk test at 95 %. The main effects of N and water shortage and leaf rank and their interactions were assessed by multifactorial analysis of variance (MANOVA), and means were compared with the Student’s *t* test (for two means) or the Tukey HSD (honestly significant difference) post hoc test (for more than two means). When the distribution was not normal, the non-parametric Mann–Whitney *U* test (to compare two ranks) or Kruskal–Wallis *H* test (to compare more than two ranks) was used. Correlations were revealed through Spearman’s rank correlation coefficient analysis. Minimal statistical significance was postulated at *P* ≤ 0.05.

## Results

### Phenology, physiology and water status of oilseed rape under restricted nitrogen and/or water supply during vegetative growth

To appreciate the impact of N and water availability on the global dynamics of oilseed rape growth, we first defined an experimental time course within the vegetative stage of plant development where watering was stopped on day 0, with different nitrate treatments having started 2 weeks before this (Fig. 1). Half of the plants receiving high or low N input were deprived of water for 2 weeks and then irrigated as normal for 8 days when the experiment ended.

**Fig. 2:**
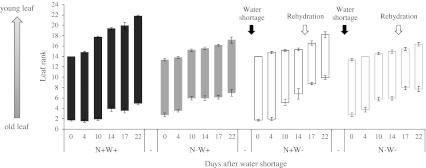
Phenological response of oilseed rape plants subjected to nitrogen depletion and/or water shortage as estimated by the number of leaves per plant during treatments. Treatments are referred to as described in Fig. 1. The oldest leaves were ranked #1 and were taken into account when their petiole was still attached to the stem. Cotyledons were not considered. The youngest leaves were counted when their petiole became visible. The total leaf number was obtained by difference between the youngest and the oldest leaf ranks. *Values* are expressed as means of five independent plants ± standard error

To initially assess leaf development and senescence in the different N and water conditions, the total number of newly emerged, attached and fallen leaves were counted at each sampling date (Fig. 2). Control plants (fully N-supplied and watered, N+W+) had an average of 12 leaves after 7 weeks of vegetative growth (i.e. on day 0), reaching a total of 17 leaves at the end of the experiment (day 22). Both nitrogen (N–W+) and water (N+W−) depletion slowed young leaf development and accelerated mature leaf senescence and abscission, as fewer leaves emerged and more leaves fell compared with control plants. As a result, these plants had fewer leaves on day 22, 10.2 leaves for N−W+ and 8.3 leaves for N+W−. The phyllochrons of N−W+ and N−W− plants were quite similar so water shortage did not have an additional impact on the development of plants which were already N-deficient. Moreover, as illustrated in Online Resource 1, visual symptoms of wilting were far less pronounced in N−W− plants than fully N-supplied plants (N+W−) after 2 weeks of water shortage, even though the latter appeared to recover better during rehydration.

To complement these phenological observations, a wide range of water status and other physiological parameters were measured in leaves at four characteristic developmental stages. A “senescent” leaf rank (#6) refers to leaves with a maximum surface area showing early indications of yellowing at day 0; a “mature” leaf rank (#9) fully developed during the first days of the experiment before initiating senescence; a “young” leaf rank (#12) had already appeared on day 0 and continued to grow during the experimental time course; an “emerging” leaf rank (#15) refers to a leaf that appeared during the experiment from day 14 onwards. Particular attention was paid to day 14 of the experiment as this was when effects of the treatments differed most clearly and to leaf ranks #9 and #12 as these showed the greatest differences between treatments throughout the experiment.

**Fig. 3:**
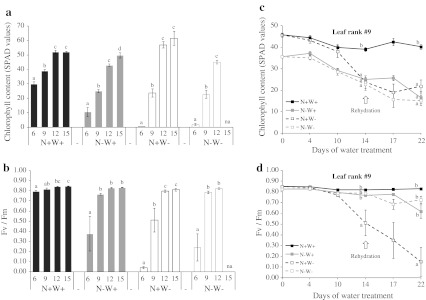
Changes in chlorophyll content (**a**, **c**) and maximum photosynthetic efficiency (*F*_v_/*F*_m_; **b**, **d**) in leaves of oilseed rape plants with high or low N input with or without water shortage (N+W+, *line with black filled square*; N−W+, *line with grey filled square*; N+W−, *line with* *open square*; N−W−, *line with grey* *open square*; treatment codes as in Fig. 1). **a**, **b** Results shown are for leaf ranks #6, #9, #12 and #15 after 14 days of treatment (day 14). **c**, **d** Results for leaf rank #9 during the 22 days of the experiment. *Values* are expressed as means of five independent replicates ± standard error. *Different letters* indicate significant differences in the Tukey HSD test (*P* ≤ 0.05) between leaf ranks (**a**, **b**) or treatments after 14 and 22 days of treatment (**c**, **d**). *na* Leaf not apparent

Foliar senescence was monitored indirectly by measuring changes in the chlorophyll content and maximum photosynthetic efficiency (*F*_v_/*F*_m_) of leaves (Fig. 3). On day 14, regardless of the N and water supply, chlorophyll content and *F*_v_/*F*_m_ to a lesser extent differed within leaf ranks, increasing in a gradient from the older to the younger leaves (Fig. 3a, b; Online Resource 2). Compared with control leaves, N deficiency (N−W+) significantly lowered chlorophyll content in all leaves sampled (Fig. 3a, c) and lowered *F*_v_/*F*_m_ in the two oldest leaf ranks (for example, −35.8 and −6.3 % for leaf #9 at day 14) (Fig. 3b, d). Water deficit (N+W−) only strongly affected the chlorophyll content and *F*_v_/*F*_m_ of #6 and #9 leaves (e.g. −39.1 and −37.4 % for leaf #9 at day 14). The losses in chlorophyll content and photosynthetic efficiency caused by water shortage did not occur in N-deprived plants (N−W−), except for leaf #6. These differences between control and treated leaves were sustained for the time course of the experiment even during the rehydration phase (Fig. 3c, d).

**Fig. 4:**
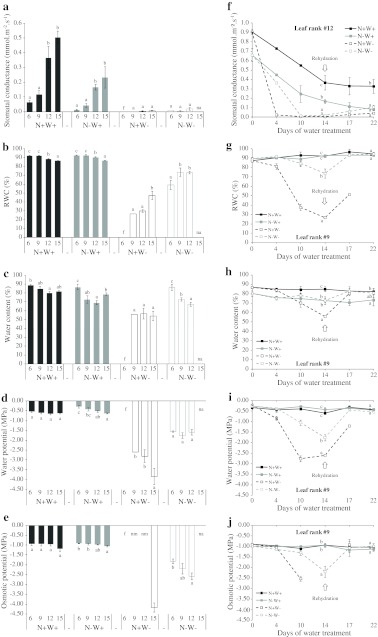
Changes in stomatal conductance (**a**, **f**), relative water content (RWC, **b**, **g**), water content (**c**, **h**), water potential (**d**, **i**) and osmotic potential (**e**, **j**) in leaves of oilseed rape plants receiving high or low N input with or without water shortage (N+W+, *line with black* *filled square*; N−W+, *line with grey* *filled square*; N+W−, *line with* *open square*; N−W−, *line with grey* *open square*; treatment codes as in Fig. 1). **a**–**e** Results for leaf ranks #6, #9, #12 and #15 after 14 days of treatment (day 14). **f** Results for leaf rank #12. **g**–**j** Results for leaf rank #9 during the 22 days of the experiment. *Values* are expressed as means of five independent replicates ± standard error. *Different letters* indicate significant differences in the Tukey HSD test (*P* ≤ 0.05) between leaf ranks (**a**–**e**) or treatments after 14 and 22 days of treatment (**f**–**j**). *f* Leaf fallen, *na* leaf not apparent, *nm* not measured as not possible to collect leaf juice

Stomatal conductance (*g*_s_), a major attribute of water management, was measured in leaves responding to N and water treatments (Fig. 4a, f). Whatever the treatment, *g*_s_ decreased with increasing leaf age. N deficiency led to lower *g*_s_ in all leaves in comparison with control conditions even before water deprivation (for example, 0.66 and 0.91 mmol m^−2^ s^−1^ in leaf #12 at day 0, Fig. 4f). In N+W− plants, water shortage caused a faster decline in *g*_s_ than in N−W− plants, with stomata being completely closed on day 10. In addition, stomata did not fully reopen during the 8 days of rehydration.

In terms of leaf water status, most of the parameters measured stayed relatively stable at the optimum level in all leaf ranks in well-watered plants (N+W+ and N−W+) throughout the experiment. Specifically, values were 91.0 ± 0.7 % for relative water content (RWC), 84.2 ± 0.8 % for water content (WC), −0.43 MPa ± 0.03 for water potential (Ψ_w_) and −0.98 MPa ± 0.03 for osmotic potential (Ψ_S_) (Fig. 4). N depletion only affected the WC of middle rank leaves with a sustained 11.5 % reduction (Fig. 4c, h). Under drought stress, all water status parameters fell to very low values. Leaves of fully N-supplied plants (N+W−) responded differentially to dehydration, observed through the progressive fall in water potential both in older and newly formed leaves. For example, leaf #15 showed a minimum Ψ_w_ of −3.83 MPa compared with −2.60 MPa for leaf #9 at the end of water shortage (Fig. 4d). Effective properties of osmotic adjustment could be appreciated in N+W− plants (leaf #15) as the fall in osmotic potential was only partly explained by water loss (Fig. 4e). For all leaf ranks tested, water status in N-deficient plants was much less affected by water deprivation than in N-supplied plants. Indeed, in leaf #9 of N−W− plants after 14 days of water shortage, values of 73.7 % for RWC, 72.9 % for WC and −1.76 MPa for Ψ_w_ were recorded in comparison with, respectively, 26.8, 56.0 % and −2.60 MPa in N+W− plants. RWC was shown to be statistically the most sensitive parameter with which to evaluate the impact of the drought treatment (Online Resource 2). Despite the duration and severity of the drought treatment which caused the loss of older leaves (while equivalent leaves were still attached to control plants), water-stressed plants were able to recover rapidly as confirmed by the individual variables. For N−W− plants, the same values as those of control plants were recovered after 3 days of rehydration.

### Whole plant proline response to restricted nitrogen and/or water supply

Proline is a nitrogenous osmolyte known to play a role in plant stress tolerance as it accumulates to high levels in response to osmotic and many other abiotic stresses. As proline metabolism could impact leaf N utilization and remobilization, especially under N deficiency, we appraised how oilseed rape proline metabolism responds to water starvation in different conditions of N availability.

**Fig. 5:**
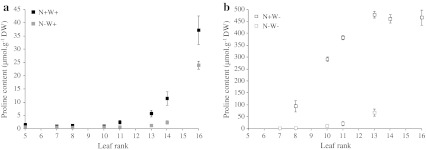
Free proline content of leaves of oilseed rape plants with high or low N input (N+W+, *black* *filled square*; N−W+, *grey* *filled square*, **a**) followed or not by water shortage (N+W−, *open square*; N−W−, *grey* *open square*, **b**) after 14 days of treatment. *Values* are expressed as means of five independent replicates ± standard error

**Fig. 6:**
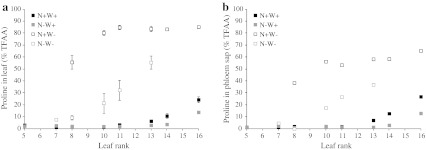
Proline as a percentage of total free amino acids (TFAA) in laminae (**a**) and phloem sap (**b**) of leaf ranks of oilseed rape plants with high or low N input (N+W+, *black* *filled square*; N−W+, *grey* *filled square*) followed or not by water shortage (N+W−, *open square*; N−W−, *grey* *open square*) after 14 days of treatment. *Values* are expressed as means of five independent replicates ± standard error

In well-watered plants, the leaves of which showed no signs of water stress (see above), proline clearly accumulated preferentially in young and newly emerged leaves in an N-dependent manner (Fig. 5a). Proline content reached 37.2 μmol g^−1^ DW in leaf #16 of N+W+ plants on day 14. Proline was thus the major free amino acid present, representing 24.2 % of total free amino acids (TFAA) while glutamate and glutamine represented 15.5 and 10.2 %, respectively. Regardless of leaf rank, proline levels (proline content and percentage of TFAA) were lower when N was limiting (Figs. 5a, 6a).

During water stress, proline was also much more abundant under the N-rich regime. In N+W− plants, proline content progressively increased from old to young leaves, reaching a maximum in leaf #13 (479 μmol g^−1^ DW) after 14 days of water shortage (Figs. 5b, 6a). Proline accounted for up to 83.6 % of TFAA and about 5.5 % of total leaf dry weight for leaf #13. By comparison, in N−W− plants, despite a similar gradient, proline accumulation was relatively slight (maximum 67 μmol g^−1^ DW and 55.4 % of TFAA obtained in leaf #13 on day 14).

**Fig. 7:**
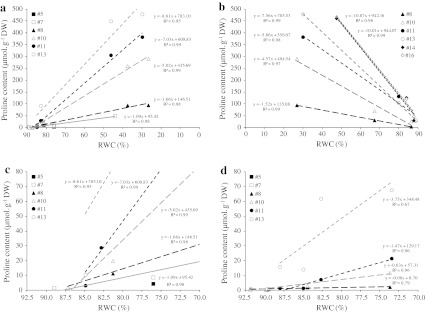
Linear relationships between free proline content and relative water content in leaves of N+W− (**a**, **c**) and N−W− (**d**) oilseed rape plants subjected to water shortage during 14 days (day 0–14), and followed by rehydration of 8 days (day 14–22) for N+W− plants (**b**). *Values* are expressed as means of five independent replicates

Combining all treatments, negative correlations between proline levels and Ψ_w_ or Ψ_S_ were somewhat reliant on leaf rank (from −0.106 in leaf #5 to −0.630 in leaf #13 for Ψ_S_), but proline content was most strongly negatively correlated with RWC (Online Resource 3). This means that at an equivalent RWC, a young leaf accumulates more proline than an older one, and a slight decrease in young leaf RWC induces a sharp accumulation of proline (Fig. 7a, d). In the same way, still considering the slopes of proline contents versus RWC, proline differently accumulated between N+ and N− plants for an equivalent decrease in RWC, e.g. by 7.0 and 1.5 μmol g^−1^ DW per % decrease in leaf #11, respectively (Fig. 7c, d).

Besides deposition, the contribution of proline to long-distance carbon and N transport was evaluated by quantifying proline in phloem sap. The proportion of proline as a percentage of TFAA in the phloem was quite similar to that in leaf laminae (Fig. 6b). Proline was the predominant amino acid in the phloem of emerging leaves in well-watered plants and of all leaves in water-stressed plants except for the two oldest leaf ranks in N−W− plants.

Finally, recovery from drought stress after watering induced a progressive decline in proline levels in all leaves and was more rapid where proline had been more abundant. On the basis of RWC, proline mobilization efficiency appeared to be slightly lower than proline accumulation efficiency (about −13.3 % whatever the leaf rank; Fig. 7b). However, after 8 days of rewatering, the pool of proline had not been completely remobilized by the plants.

In summary, proline accumulation that differs according to the leaf developmental stage (from senescing to emerging leaves) and the N fertilizer level and proline abundance in phloem sap suggest that proline might play a key role in N utilization efficiency and source-sink relationships in oilseed rape.

### Leaf capacity for proline synthesis and degradation under restricted nitrogen and/or water supply

Differential regulation of proline deposition according to leaf age could result from differential capacities for proline synthesis, degradation and/or phloem export. To evaluate the intrinsic capacity of leaf tissues to accumulate proline, leaf discs excised from control, N-deficient, water-stressed and N-deficient water-stressed plants were subjected in vitro to hyper-osmotic stress (16 h at −2.5 MPa). Using leaf lamina explants essentially creates a “closed” leaf system disconnected from the source-sink relationships within the plant, in which proline is neither supplied nor exported. The aim of creating an artificial hyper-osmotic shock was both to rapidly stimulate proline production in isolated tissues, as already demonstrated by Larher et al. (1993), and to expose leaf explants to the same external Ψ_W_.

**Fig. 8:**
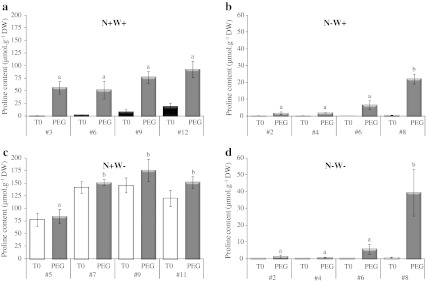
Apparent proline biosynthesis capacity of explants from distinct leaf ranks of oilseed rape plants receiving high or low N input for 10 days followed or not by water shortage for 7 days. Treatment codes are as for Fig. 1. Proline content was measured at time of sample collection (T0) and after 16 h of hyper-osmotic stress at −2.5 MPa (PEG-induced). **a** Results were obtained for leaf ranks #3, #6, #9 and #12 for control plants (N+W+). **b** Results for leaf ranks #2, #4, #6 and #8 for N-deprived plants (N−W+). **c** Results for leaf ranks #4, #7, #9 and #11 for water-stressed plants (N+W−). **d** Results for leaf ranks #2, #4, #6 and #8 for N− and water-deprived plants (N−W−). *Values* are expressed as means of four independent replicates ± standard error. *Different letters* indicate significant differences using the Tukey HSD test (*P* ≤ 0.05) between leaf ranks after PEG incubation

Leaf tissues exhibited a variable endogenous capacity for proline accumulation depending on leaf rank and N regime, while it was expected that proline accumulation should have been exacerbated in all conditions. In control plants (N+W+, Fig. 8a), proline production and accumulation were efficient in all osmotically treated leaves. Nevertheless, the proline content of explants from young leaves (93 μmol g^−1^ DW in leaf #12) was noticeably less than of leaves from whole plants after 2 weeks of water shortage (478 μmol g^−1^ DW in leaf #13). In water-stressed plants (N+W−, Fig. 8c), the osmotic treatment was not sufficient to generate additional accumulation of proline in leaves, probably because of their already reached very low Ψ_W_. These results show that the chosen greenhouse conditions tended to create a strong water-deficit stress. In N-deficient plants (N−W+ and N−W−, Fig. 8b, d), proline content created by the hyper-osmotic shock increased in a gradient from older to younger leaves, but the capacity to over-accumulate proline was still less efficient than in control plants, e.g. there was only 22.2 μmol g^−1^ DW of proline in leaf #8 of N−W+ plants after 16 h at −2.5 MPa, possibly because nitrogenous substrates for proline metabolism were limited (Fig. 8b).

As it is possible that a lack of proline metabolic precursors limited the response to osmotic shock, a similar experiment was carried out on leaf explants over-enriched with glutamine (40 mM) for 6 h before the osmotic treatment (Online Resource 4b). When exogenous glutamine was supplied in this way there was a significant increase in the amount of proline that accumulated in response to osmotic shock, for example, 198.40 μmol g^−1^ DW in leaf #3 of N+W+ plants (Online Resource 4a) compared with 56.53 μmol g^−1^ DW without glutamine enrichment (Fig. 8a). The proline production capacity of N-deficient leaves was still lower than those of control N-supplied leaves and still higher in the youngest leaves regardless of the N and water treatment (Online Resource 4a).

**Fig. 9:**
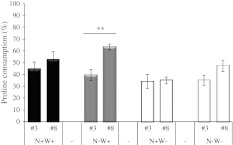
Apparent proline consumption capacity of explants from leaf ranks #3 and #8 of oilseed rape plants receiving high or low N input for 10 days (N+W+ *black* *filled square*/N−W+ *grey* *filled square*) followed or not by water shortage for 7 days (N+W− *open square*/N−W− *grey* *open square*). The percentage of proline consumed after 4 hours in −0.04 MPa medium was estimated relative to the proline content measured after 6 hours of enrichment with proline (40 mM). *Values* are expressed as means of five independent replicates ± standard error. *Asterisks* indicate significant differences between leaf ranks for each treatment in the Student’s *t* test (*P ≤ 0.05; **P ≤ 0.01; ***P ≤ 0.001)

To assess the intrinsic capacity of leaves for proline degradation in terms of leaf rank and treatment, leaf explants were enriched with exogenous proline (40 mM) for 6 h before incubation on iso-osmotic medium (−0.04 MPa) for 4 h (Fig. 9, Online Resource 5). Results showed that only N deficiency increased the capacity for proline consumption in the younger leaves whereas there was no significant difference between leaf ranks when well supplied with N.

**Fig. 10:**
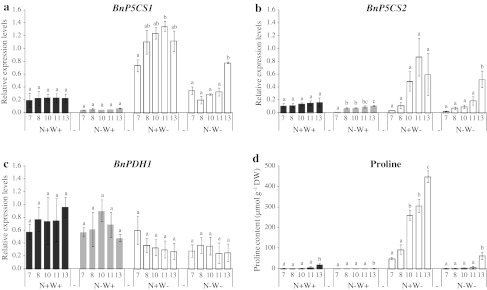
Relative expression levels measured by qRT-PCR of *BnP5CS1* (**a**), *BnP5CS2* (**b**) and *BnPDH1* (**c**) genes and free proline contents (**d**) in leaf ranks #7, #8, #10, #11 and #13 of oilseed rape plants grown with high or low N input for 14 days (N+W+ *black* *filled square*/N−W+ *grey* *filled square*) followed or not by water shortage for 10 days (N+W− *open square*/N−W− *grey* *open square*). *Values* are expressed as means of three independent replicates ± standard error. *Letters* indicate significant differences in the Tukey HSD test (*P* ≤ 0.05) between leaf ranks for each treatment

To explore the molecular basis of these contrasting proline regulation responses, the expression levels of members of two major gene families involved in proline metabolism were studied. Relative transcript levels of two members of the biosynthetic *BnP5CS* genes (*BnP5CS1* and *BnP5CS2*) and the *BnPDH1* proline oxidation gene were estimated using qRT-PCR in plants on day 10 of the water shortage period (Fig. 10). Free proline content and *P5CS1* and *P5CS2* expression levels varied in similar patterns in terms of plant development and physiology (Fig. 10a, b, d), since differences in proline content were significantly correlated with changes in *P5CS1* and *P5CS2* gene transcript levels (0.872 and 0.862, *P* < 0.001) with respect to leaf age and treatment. However, whereas the proline content and the capacity of proline accumulation increased gradually from older to younger leaves, the gradients of expression of the *P5CS* genes were less pronounced, without evidence of specific over-induction in the youngest leaves, particularly in N+W− leaves that were the most enriched in proline.

Although they were strongly correlated with each other (0.816, *P* < 0.001), *P5CS1* and *P5CS2* expression profiles were slightly different (Fig. 10a, b). *P5CS1* was generally expressed the most and also appeared to be the water stress-induced isoform (Online Resource 6) as water deprivation significantly induced expression of *P5CS1*, a response that was much less pronounced for *P5CS2*. For example, *P5CS1* expression was 5.0-fold higher in leaf #13 of plants that had been water-stressed for 10 days than in watered plants (whereas *P5CS2* expression was 3.7-fold higher; Fig. 10a, b). Conversely, N depletion significantly inhibited *P5CS1* expression only (Online Resource 6), which probably explains the lower level of proline accumulation in all N-deficient leaves compared with controls.

Concerning proline oxidation, *BnPDH1* expression was negatively correlated with those of *P5CS1* and *P5CS2* (−0.407 and −0.442), confirming their antagonistic regulation and with proline content (−0.350) although not at a significant level. Unlike proline content, *PDH1* expression was relatively uniform along the plant axis whatever the treatment (Fig. 10c). In addition, the strong accumulation of proline during drought was not correlated with a significant decrease in *PDH1* expression levels, and the fall in proline content under N depletion was not associated with an enhancement in *PDH1* transcription (Online Resource 6).

## Discussion

In the near future, field-grown winter oilseed rape may be required to frequently withstand both restricted N availability due to the introduction of low N input crop management practices and limited water supply due to forecast climatic changes. Current oilseed rape breeding programmes are seeking better NUE genotypes adapted to limited N conditions but do not necessarily take into account the impact that such adaptations might have on the plant’s tolerance to other environmental constraints. Here the effect of watering was assayed in plants that had received high or low amounts of N at the rosette stage, a critical stage for nutrient source-sink remobilization between leaves. The aim was to examine the influence of N status (1) on plant survival and recovery from a strong water deficit and (2) on the efficiency of accumulation and remobilization of proline along the plant axis (by testing individual leaf ranks) in terms of it being both a well-known stress osmolyte and a putative N source metabolite. How much does proline metabolism under water stress contribute to nitrogenous osmolyte deposition in leaves for osmotic adjustment and/or how much to N-nutrient translocation from source to sink leaves, especially when N-fertilizer is limited? Is there any physiological antagonism between these two roles? Oilseed rape is an ideal species in which to reliably test such relationships as it is an extremely potent accumulator of proline under water stress and knowledge of the metabolic attributes of N remobilization could contribute to improving NUE.

### Chlorophyll content and RWC are the most reliable parameters to reflect leaf development and growth conditions under different N and water regimes

As known in many plant species, we showed that leaf fall in oilseed rape can be prematurely induced and accelerated by adverse environmental conditions, in this case restricted N and water availability (Figs. 2, 3; Masclaux-Daubresse et al. 2008). Senescence was monitored through the changes in chlorophyll content (as by Buchanan-Wollaston 1997) and maximum photosynthetic efficiency in leaves (as by Baker and Rosenqvist 2004; Woo et al. 2008). In control plants (fully N-supplied and watered), leaf senescence was mainly indicated by a drop in chlorophyll content, whereas the quality and the integrity of photosystem II remained near intact as the leaves aged (Fig. 3; Online Resource 7). Wingler et al. (2004) showed that visible leaf senescence in *A. thaliana* correlated well with a decline in *F*_v_/*F*_m_, although according to Woo et al. (2008) the decline was too late and sudden. However, it was found that drought does not modify *F*_v_/*F*_m_ in many plant species, such as potato (Jefferies 1994) or soybean (Ohashi et al. 2006), and N supply has to reach very low levels before *F*_v_/*F*_m_ is affected in apple leaves (Cheng et al. 2000).

In our experiment, leaf rank was statistically the principal factor in the variability of chlorophyll content and maximum photosynthetic efficiency (Online Resource 2). Of the two parameters, chlorophyll content was the most sensitive to leaf age and environmental factors (*R*² = 0.94) and its changes were strongly correlated (0.918, *P* < 0.001) with the activity of the photosynthetic apparatus (Online Resource 7). Thus, chlorophyll content could be used as an indicator of leaf age and development, particularly as it was more readily measured than the maximum photosynthetic efficiency.

In the same way, by comparing different water status parameters it was possible to determine the ones which could be best used to screen for drought stress or N deficiency during vegetative growth (Fig. 4). Some indices have already been used to screen for drought tolerance in *Brassica* species but at later stages of development in oilseed rape (Pasban Eslam 2009).

Our results demonstrated that all water status parameters varied not only with water treatment as expected, but were also considerably affected by N regime and leaf age (Online Resource 2). Moreover, the effect of water stress clearly depended on N status, and the corresponding interactions linked with leaf rank implied that leaves were indeed differently sensitive to contrasting conditions. RWC seemed to be the most sensitive to environmental perturbations and was clearly less impacted under N deprivation. These findings underline the importance of working out an experimental method with which to vary the water status homogeneously in control and N-deficient plants, perhaps by using different durations or levels of water restriction.

### Nitrogen deficiency leads to a lower sensitivity to water stress

Under our experimental conditions, all leaves were subject to dehydration during the drought period, and the youngest sampled leaf (#15) of N-supplied plants even reached the critical value of −3.83 MPa for Ψ_w_ while the plants survived (Fig. 4d). Growth observed during rehydration, as previously noted by Müller et al. (2010), shows the huge potential of oilseed rape plants to compensate for the effects of water stress, particularly when well supplied with N.

A major finding was that N limitation (0.4 mM vs. 8 mM NO_3_^−^) led to oilseed rape being less sensitive to water stress over the short time scale experienced in our study (i.e. in the context of the entire whole growth cycle). As development was affected in terms of the total number of leaves (Fig. 2; Online Resource 1), N-deprived plants were far from experiencing dehydration at a comparable level to N-supplied plants during the drought period (Online Resource 1; Fig. 4), clearly exhibiting a low Ψ_w_ stress avoidance response (Verslues et al. 2006). Although N fertilizers and irrigation are known to separately increase grain yield in oilseed rape (Kamkar et al. 2011), Andersen et al. (1996) noticed that yield when water was withdrawn can be slightly higher under low N nutrition.

N availability clearly modified the morphological and physiological impact of water stress, and water requirement before shortage also differed according to the level of N nutrition. Compared with fully N-supplied plants, N-deprived plants appeared to take up and consume less water throughout the experiment as they grew more slowly and produced less biomass as fewer stems and leaves developed (as also described by Leleu et al. 2000). Photosynthetic activity and stomatal conductance were both lower in leaves of N-deprived plants (Gammelvind et al. 1996) and this has been linked to a retro-inhibitory regulation of the carbon/nitrogen balance (Wingler et al. 2004).

The partial stomatal closure in leaves of N-deficient plants observed on day 0 (Fig. 4f) would partly explain the less pronounced impact of drought. This would limit water loss by transpiration and protect leaves against dehydration, at least over a relatively short drought period. A similar lower stomatal conductance in N-limited plants has been noted in lettuce and spring wheat (Broadley et al. 2001; Li et al. 2004). Some information concerning the role of mineral nutrition in alleviation of drought stress has been recently reviewed (Waraich et al. 2011); however, signalling events that link stomatal regulation to plant nitrogen status remain to be elucidated.

### Proline accumulation preferentially occurs in young leaves and is affected by N limitation

Our results indicated that in oilseed rape, proline accumulates in the absence of water stress on its own accounting for almost 25 % of the TFAA pool in emerging leaves of watered plants (Figs. 5a, 6a). An increasing gradient in proline content has already been distinguished from vegetative (roots and leaves) to reproductive organs (flowers and seeds) in the absence of stress in oilseed rape, *A. thaliana* and *Medicago truncatula* (Chiang and Dandekar 1995; Armengaud et al. 2004; Xue et al. 2009). Our results also demonstrate that nitrate starvation had a negative impact on proline abundance in all leaves (Online Resource 2). It may be inferred that proline accumulation can be induced without water stress signal and that stress-independent proline production efficiency may directly reflect N and metabolic substrate availability. As proposed by Sánchez et al. (2001) in green bean plants, proline could be an indicator of excess N. The presence of high levels of proline in developing and newly emerging leaves could be a form of managing excess N and represent both a reliable N resource and osmotic (and possibly energetic) generator for growth. In mature leaves, low proline levels could constitute a signal for triggering leaf senescence. Besides, the biological functions of proline in plant growth and development independent of the water stress context are still not completely clear as discussed in recent reviews (Lehmann et al. 2010; Verslues and Sharma 2010).

Otherwise, proline is thought to be a drought-associated metabolite in oilseed rape, mainly because it accumulates to much higher levels in response to drought (Online Resource 2). After 14 days of water shortage, proline reached nearly 500 μmol g^−1^ DW accounting for about 85 % of TFAA and 14 % of the total N pool in the youngest leaves of fully N-supplied plants (Figs. 5b, 6a). To our knowledge, few cultivated glycophytic species are known to contain so much proline in their leaves, an exception being Andean potatoes that accumulate proline to around 1,300 μmol g^−1^ DW (Schafleitner et al. 2007). A steeper proline content gradient from roots to upper leaves has also been described in tobacco after 6 days of strong water stress (Dobrá et al. 2011). The proline content in leaves may increase in plants experiencing not only drought, but also salinity, freezing, high light, extreme temperatures or heavy metal toxicity (Szabados and Savouré 2010).

At an equal level of leaf dehydration, more proline accumulated in the youngest leaves than in the oldest (Online Resource 3; Fig. 7). Under stressful conditions, proline accumulation is thought to play multiple positive roles in stress tolerance and plant protection, such as increasing cellular osmolarity to maintain turgor, buffering redox potential, stabilizing subcellular structures and preserving macromolecules (like proteins or DNA) (Van Rensburg et al. 1993) or scavenging reactive oxygen species (Matysik et al. 2002). It may be hypothesized then that proline acts as a protective osmolyte and chaperoning compound in the newly formed leaves of oilseed rape during drought.

A clear discrimination was made here between N-fully supplied and N-deprived plants in terms of proline production efficiency under watering challenge. As already stated, N-limited plants were able to avoid low Ψ_w_ stress and this should be mainly the cause of restriction of the stress-regulated proline metabolism. Nevertheless, when comparing the performance for proline accumulation of leaf tissues from N+ and N− plants at the same RWC, N-limited tissues remained less efficient (Fig. 7c, d). Moreover, when leaf explants were treated with PEG and were supposed to be under a similar osmotic strain, tissues from N-deprived plants were far less productive in terms of proline accumulation even if supplied with exogenous glutamine (Online Resource 4). So taken together, N limitation surely negatively impacts proline production efficiency not only through a water stress-induced response avoidance but also through an additive regulation which directly depends on N availability. Evidence is given here that glutamine acted as a very efficient substrate to boost proline production in vitro assays and it can be extrapolated that *in planta* the glutamate/glutamine cycle should be actively mobilized towards proline production. Glutamine synthetase has already been described to play a major role in controlling proline production (Brugière et al.^1999^; Díaz et al. 2010).

### Water stress-induced proline metabolism could play a significant role in N management and remobilization in leaves

Whatever the growth conditions, a differential proline metabolism pattern is associated with leaf age and source-sink status. In “drought-sensitive” plants (N+W−), where proline contents reached their peak, there were concomitant significant increases in the contents of other amino acids, like histidine, valine, tryptophan and leucine (Online Resource 9). Conversely, as in *Lotus corniculatus* (Díaz et al. 2005) and *Petunia hybrida* (Yamada et al. 2005), proline accumulation was associated mainly with depletion of glutamate, serine, aspartate and α-alanine (and glutamine in the youngest leaves only), the predominant amino acids in leaves before stress was applied. These close correlations were attenuated in N−W− plants. Proline accumulation at the expense of the predominant transport forms of N in oilseed rape (Lohaus and Moellers 2000) could interfere with remobilization by immobilizing a significant proportion of N resources in leaves during drought. Nonetheless, contents of the phloem sap clearly suggest that proline is efficiently transported, particularly in the youngest leaves (Fig. 6b). Proline accumulation in leaves was also shown to be reversible (Fig. 7b) as the rehydration phase led to a remarkable decline in proline levels similar to data from Good and Zaplachinski (1994). Possibly oxidation of proline pools could supply C and N substrates and reducing potential for mitochondria providing electrons for the respiratory chain during recovery from drought (Szabados and Savouré 2010).

As N is mainly translocated in the form of amino acids via the phloem (Feller and Fischer 1994) and N remobilization from leaf to leaf was found to be efficient in oilseed rape plant during vegetative growth (Malagoli et al. 2005), proline in phloem should play a role in N remobilization efficiency from senescing source leaves to developing sink leaves, especially when N was not limiting during water shortage. Knowing that phloem loading of amino acids is not limiting for N remobilization in oilseed rape (Tilsner et al. 2005), we focused on the intrinsic capacity of leaf tissues to produce or degrade proline. Thus, experiences using isolated leaf explants allowed us to show that both the proline biosynthesis and degradation capacities differed markedly with leaf age and N availability (Figs. 8, 9; Online Resources 4, 5). Greater accumulation in the youngest control leaves was attributed to a higher capacity to synthesize proline, while the capacity to metabolise proline was quite similar in old and young leaves. Nevertheless, the oldest leaves were able to produce proline and a significant transport flux may occur from senescing to emerging leaves (Fig. 6b). Conversely, the lower proline contents observed during N deficiency (N−) were attributed mostly to a lower capacity for synthesis even if tissues may suffer a lack of N substrate.

At the molecular level, the two families of genes involved in proline metabolism are subject to very tight transcriptional regulation. The proline biosynthesis pathway has generally been described as being up-regulated during dehydration and its catabolism down-regulated, leading to proline accumulation, whereas rehydration triggers regulation in the opposite sense (Xue et al. 2009, in *Brassica napus*). However, this paradigm has been challenged by recent studies proving that proline catabolism is not necessarily repressed at low Ψ_w_ (Sharma et al. 2011).

Two homologous *P5CS* genes coding for the rate-limiting step enzymes of proline biosynthesis have been identified in *A. thaliana*, *Medicago truncatula*, *Oryza* *sativa* and other species (Strizhov et al. 1997; Hien et al. 2003; Armengaud et al. 2004). More recently, two closely related *PDH* genes have been found to encode proline degradation enzymes in *A. thaliana* and *Nicotiana* *tabacum* (Ribarits et al. 2007; Funck et al. 2010). Both *P5CS* and *PDH* isoforms have non-redundant physiological functions in plant development and stress tolerance, commonly being differentially regulated as a housekeeping form and a stress-specific form. The specific role and regulation of each paralog vary from one species to another. For example, *AtP5CS2* and *MtP5CS1* expression are consistent with a housekeeping function, and proline accumulation under osmotic stress is primarily contingent upon induction of *AtP5CS1* or *MtP5CS2* expression (Strizhov et al. 1997; Armengaud et al. 2004; Székely et al. 2008). Equally, while *AtPDH2* was up-regulated and *NtPDH1* little affected by osmotic stress, *AtPDH1* and *NtPDH2* expression were inhibited (Funck et al. 2010; Dobrá et al. 2011).

*BnP5CS1*, *BnP5CS2* and *BnPDH1* genes were studied here by qRT-PCR (Fig. 10). Only one *PDH* homolog was found in oilseed rape by searching with *AtPDH1* and *AtPDH2* sequences. Free proline levels in leaves coincided well with expression patterns of the two *BnP5CS* genes, which were similarly regulated: induced by water deficit as expected, and interestingly inhibited during N deficiency. However, unlike previous reports (Armengaud et al. 2004; Xue et al. 2009), *BnP5CS1* was both the most highly expressed isoform in leaves under normal growth conditions and the most affected by environmental constraints. So *P5CS1* transcriptional regulation seems to play an important role in proline biosynthesis in oilseed rape. Induced levels of *P5CS* expression were lower than the fold increase in proline during water stress (an average of five- and threefold higher for *P5CS1* and *P5CS2* expression, respectively vs. 70-fold higher proline content in leaves of water-stressed plants relative to watered controls). These values were also lower than those obtained by Xue et al. (2009) in oilseed rape after osmotic stress (maximum 132-fold and 33-fold for *BnP5S1* and *BnP5CS2* after 12 h of PEG treatment, with a maximum of 25-fold proline accumulation after 48 h). *P5CS* induced expressions did not vary noticeably in terms of leaf position or age compared with proline accumulation (Online Resource 6), which is all the more significant as *PDH1* gene expression was not strongly modified by applied abiotic stresses or at different leaf developmental stages unlike previous reports cited above.

In addition to the slight variations in transcriptional regulation of *BnP5CS* and *BnPDH1* genes, the contrasted variations in proline level in leaf tissues of oilseed rape plants subjected to various stresses are likely to involve a complex range of processes, such as differential activities of the corresponding enzymes, mechanisms of proline remobilization and transport within and between leaves, or a putative highly regulated *BnPDH2* gene, all of which will need to be studied in more depth.

## Conclusion

The present work reveals that when mineral N is limiting at the rosette stage of oilseed rape development, the accumulation of free proline in leaves, particularly in response to a drought period, is inhibited with a reduction in the loss of internal water. Our experiments provide evidence that less proline accumulation in N-deprived, slow-growing plants is not related to a deficiency in metabolic precursors but to lower production efficiency, amplified further during drought by an attenuated response to water stress. Whatever the N and water status of the plant, proline accumulation in leaves is not equal along the plant axis with more accumulation in the newly emerged and emerging leaves. Thus, besides its role in plant stress tolerance, accumulated proline may represent a substantial reserve of substrate for growth in the form of organic N that is easily remobilized (both spatially and metabolically) to supply the youngest leaf tissues during growth and recovery from stress. Proline may be involved in the global dynamics of N remobilization in oilseed rape, either by transport, as corroborated by its high percentage in the phloem, or by utilization in leaves as an intermediary of glutamate–glutamine metabolism. Proline accumulation should therefore not be considered merely as a passive deposition of osmoticum in stressed tissues but rather as a dynamic reservoir of N and reductant which strongly impacts NUE especially under water stress but also in fully hydrated growing tissues. Limiting N fertilizer input will have global consequences on plant water relations and proline production efficiency is one aspect that can be explored in more depth to guide crop management and breeding programmes.

## Electronic supplementary material

Below is the link to the electronic supplementary material.

Supplementary material 1 (PDF 2197 kb)

## Abbreviations

DW: Dry weight
*F*_v_/*F*_m_: Maximum photosynthetic efficiency
*g*_s_: Stomatal conductance
N: Nitrogen
NUE: Nitrogen use efficiency
Ψ_S_: Osmotic potential
*P5CS*: Δ^1^-Pyrroline-5-carboxylate synthetase
*PDH*: Proline dehydrogenase
qRT-PCR: Quantitative reverse transcription-polymerase chain reaction
RWC: Relative water content
TFAA: Total free amino acids
W: Water
Ψ_w_: Water potential
