# Supplementary material for: Nitrogen availability impacts oilseed rape (Brassica napus L.) plant water status and proline production efficiency under water-limited conditions
Source: Planta. 2012 Apr 8;236(2):659–76. doi: 10.1007/s00425-012-1636-8 (PMC3404282; doi:10.1007/s00425-012-1636-8)
Supplement: Supplementary file 1 — Supplementary material 1 (PDF 2197 kb) [file 425_2012_1636_MOESM1_ESM.pdf]

Nitrogen availability impacts oilseed rape (*Brassica napus* L.) plant water status and proline production efficiency under water-limited conditions

Planta

Benjamin Albert, Françoise Le Cahérec, Marie-Françoise Niogret, Pascal Faes, Jean-Christophe Avice, Laurent Leport, Alain Bouchereau

A. Bouchereau
alain.bouchereau@univ-rennes1.fr
UMR 1349 INRA - Agrocampus Ouest - Université de Rennes 1, Institut de Génétique, Environnement et Protection des Plantes, Campus de Beaulieu, F-35042 Rennes cedex, France

Electronic supplementary material

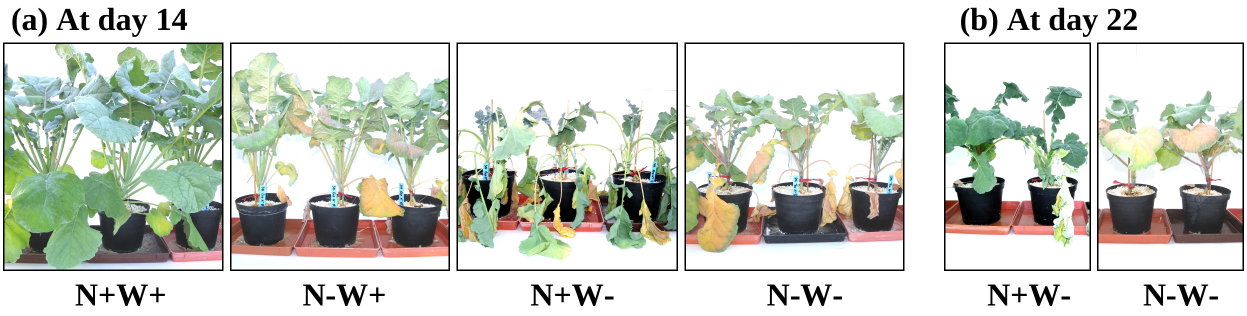

Online resource 1 Photographs of **a** 9-week-old oilseed rape phenotypes, for control plants (N+W+), N-deprived plants for four weeks (N-W+), water-stressed plants for two weeks (N+W-) and combined N- and water-limited plants (N-W-) and of **b** 10-week-old oilseed rape phenotypes, for N+W- and N-W- plants after one week of rehydration

Online resource 2 Multifactorial analysis of variance for chlorophyll content, maximum photosynthetic efficiency (F_v/F_m), relative water content, water content, water potential, osmotic potential, stomatal conductance and proline content measured on leaf ranks #6, #9, #12, #15 after 14 days of treatment, using nitrogen supply, water supply and leaf rank as main effects and all corresponding interaction effects

**Factor** / **Trait**	Nitrogen (N)	Water (W)	Leaf rank (L)	N × W	N × L	W × L	N × W × L	R^2
Chlorophyll content	157.43 ***	6.90 *	320.82 ***	0.75 NS	9.14 ***	11.22 ***	8.51 ***	0.94
Fv / Fm	3.01 NS	9.33 **	24.73 ***	4.28 *	9.17 ***	2.01 NS	1.10 NS	0.59
Relative water content	442.51 ***	1828.39 ***	7.10 ***	411.51 ***	9.40 ***	27.59 ***	7.42 ***	0.97
Water content	73.41 ***	553.93 ***	58.38 ***	348.47 ***	14.98 ***	2.31 NS	6.37 ***	0.94
Water potential	130.44 ***	745.19 ***	7.85 ***	87.59 ***	4.20 **	3.00 *	7.87 ***	0.93
Osmotic potential	266.21 ***	1464.96 ***	4.54 **	245.61 ***	2.32 NS	1.48 NS	2.77 *	0.96
Stomatal conductance	38.97 ***	242.07 ***	46.99 ***	45.27 ***	4.26 **	44.21 ***	5.95 **	0.89
Proline content	2292.73 ***	2834.69 ***	215.31 ***	2174.58 ***	105.77 ***	161.49 ***	92.76 ***	0.99

Values are statistical results obtained with Fisher-Snedecor test. NS, non significant; *, P ≤ 0.05; **, P ≤ 0.01; ***, P ≤ 0.001

Online resource 3 Correlations between free proline content, water status and physiological traits for leaf ranks #5, #7, #8, #10, #11 and #13 during 14 days of nitrogen and water treatments

**Proline content on leaf rank**	Chlorophyll content	Fv / Fm	Relative water content	Water content	Water potential	Osmotic potential	Stomatal conductance
#5	0.253 **	0.168 *	-0.583 ***	-0.298 ***	-0.357 ***	-0.106	-0.082
#7	0.059	0.005	-0.695 ***	-0.238 **	-0.447 ***	-0.147	-0.148
#8	0.174 *	-0.036	-0.695 ***	0.025	-0.563 ***	-0.232 **	-0.234 **
#10	0.127	-0.049	-0.707 ***	0.001	-0.583 ***	-0.272 ***	-0.250 **
#11	0.589 ***	-0.056	-0.688 ***	-0.059	-0.603 ***	-0.450 ***	-0.352 ***
#13	0.486 ***	-0.053	-0.706 ***	-0.174 *	-0.523 ***	-0.630 ***	-0.421 ***

Spearman coefficients. *, $P \leq 0.05$; **, $P \leq 0.01$; ***, $P \leq 0.001$

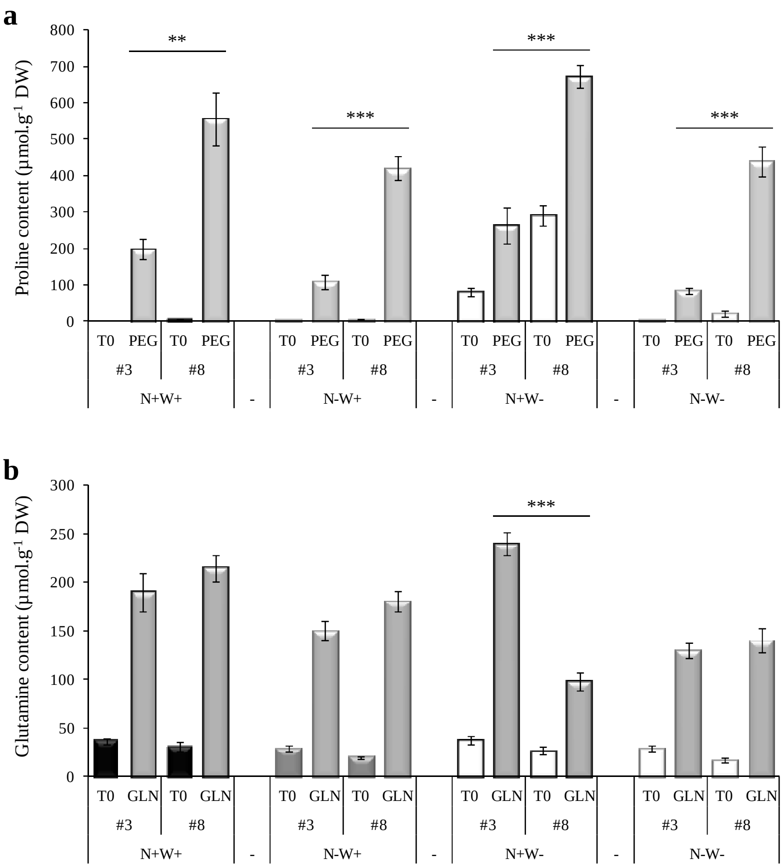

Online resource 4 Apparent proline biosynthesis capacity of **a** leaf explants enriched in glutamine from ranks #3 and #8 of oilseed rape plants with high or low N input (N+W+ / N-W+) with or without water shortage for seven days (N+W- / N-W-). The proline and glutamine contents were measured in leaf explants when sampled (T0) and after six hours of enrichment with glutamine (GLN, **b**) followed by 24 hours of hyper-osmotic stress at -2.5 MPa (PEG, **a**). Values are expressed as means of five independent replicates ± standard error. Asterisks indicate significant differences in the Student t-test (*, $P \leq 0.05$; **, $P \leq 0.01$; ***, $P \leq 0.001$) between leaf ranks for each treatment after glutamine enrichment or PEG incubation

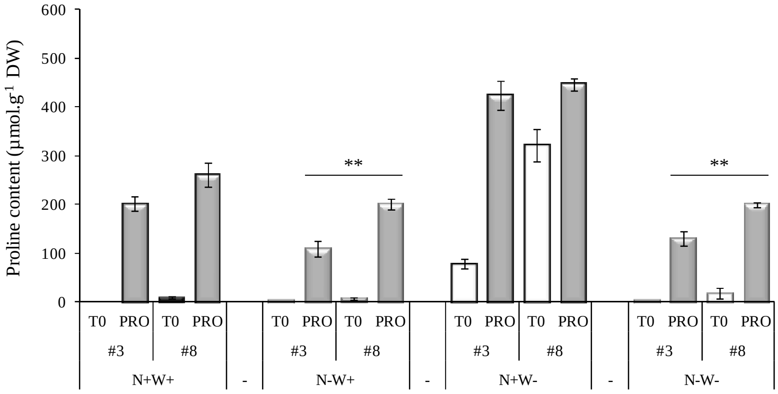

Online resource 5 Proline content in leaf explants from ranks #3 and #8 of oilseed rape plants receiving high or low N input (N+W+ / N-W+) with or without water shortage for seven days (N+W- / N-W-). The proline content was measured in leaf explants when sampled (T0) and after six hours of enrichment with proline (PRO). Values are expressed as means of five independent replicates ± standard error. Asterisks indicate significant differences in the Student t-test (*, $P \leq 0.05$; **, $P \leq 0.01$; ***, $P \leq 0.001$) between leaf ranks for each treatment

Online resource 6 Analyses of variance for the relative expression levels of the *BnP5CS1*, *BnP5CS2* and *BnPDH1* genes and proline content measured on four distinct leaf ranks after 10 days of treatment, using nitrogen supply (N), water supply (W) or leaf rank (L) as main effects in well-watered (W+) or water-stressed (W-) plants, with high (N+) or low (N-) N input, in control (N+W+) or water-stressed plants (N+W-)

**Factor** / **Trait**	N in W+	N in W-	W in N+	W in N-	L in N+W+	L in N+W-
P5CS1	7.17 *	43.20 ***	75.89 ***	24.55 ***	1.46 NS	2.77 NS
P5CS2	2.05 NS	3.82 NS	7.19 *	3.70 NS	5.87 *	2.18 NS
PDH1	0.00 NS	0.95 NS	3.41 NS	11.13 **	2.88 NS	1.03 NS
Proline	6.41 *	44.22 ***	47.98 ***	9.31 **	212.51 ***	11.02 **

Values are statistical results obtained with Fisher-Snedecor test. NS, non significant; *, P ≤ 0.05; **, P ≤ 0.01; ***, P ≤ 0.001

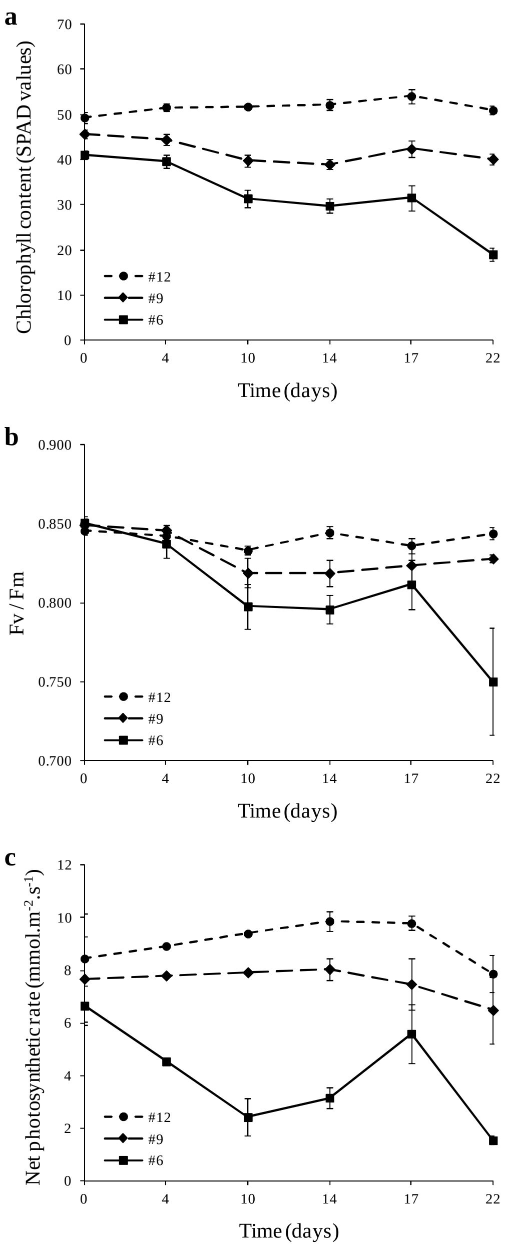

Online resource 7 Changes in **a** chlorophyll content, **b** maximum photosynthetic efficiency and **c** net photosynthetic rate in leaf ranks #6 (■), #9 (♦) and #12 (●) of control plants (N+W+) during the 22 days of the experiment. Values are expressed as means of five independent replicates ± standard error

Online resource 8 Correlations between water status and physiological traits for leaf ranks #6, #9 and #12 during 14 days of nitrogen and water treatments

**Leaf rank**	**Trait**	Chlorophyll content	Fv / Fm	Relative water content	Water content	Water potential	Osmotic potential	Stomatal conductance
#6	Chlorophyll content	1						
	Fv / Fm	0.894 ***	1					
	Relative water content	0.128	0.187 *	1				
	Water content	0.123	0.260 **	0.269 ***	1			
	Water potential	0.425 ***	0.481 ***	0.539 ***	0.393 ***	1		
	Osmotic potential	0.343 ***	0.318 ***	0.277 ***	0.386 ***	0.371 ***	1	
	Stomatal conductance	0.693 ***	0.726 ***	0.308 ***	0.402 ***	0.679 ***	0.451 ***	1
#9	Chlorophyll content	1						
	Fv / Fm	0.768 ***	1					
	Relative water content	0.137	0.236 **	1				
	Water content	0.672 ***	0.755 ***	0.252 **	1			
	Water potential	0.277 ***	0.464 ***	0.545 ***	0.381 ***	1		
	Osmotic potential	0.361 ***	0.428 ***	0.418 ***	0.550 ***	0.462 ***	1	
	Stomatal conductance	0.629 ***	0.757 ***	0.380 ***	0.723 ***	0.664 ***	0.561 ***	1
#12	Chlorophyll content	1						
	Fv / Fm	0.149	1					
	Relative water content	-0.325 ***	0.134	1				
	Water content	0.071	0.683 ***	0.269 ***	1			
	Water potential	-0.385 ***	0.346 ***	0.517 ***	0.492 ***	1		
	Osmotic potential	-0.104	0.230 **	0.576 ***	0.509 ***	0.519 ***	1	
	Stomatal conductance	-0.172 *	0.531 ***	0.353 ***	0.799 ***	0.656 ***	0.583 ***	1

Spearman coefficients. *, $P \leq 0.05$; **, $P \leq 0.01$; ***, $P \leq 0.001$

Online resource 9 Correlations between proline content and the content of other free amino acids in leaves of oilseed rape plants with high or low N input (N+W+ / N-W+) with or without water shortage (N+W- / N-W-)

Treatment	Leaf	Amino acid																										
		Glu	Gln	Asp	Asn	HO-Pro	His	SMCSO	Ser	Arg	Gly	Hser	β-Ala	Thr	α-Ala	GABA	SMC	Orn	Cys	Lys	Tyr	Met	Val	Ile	Leu	Phe	Trp	Σ AAs
N+W+	all	0.556 ***	0.683 ***	0.583 ***	0.790 ***	0.370 ***	0.247 **	0.149	0.679 ***	0.492 ***	0.138	0.742 ***	0.534 ***	0.818 ***	0.690 ***	0.149	-0.586 ***	-0.078	0.313 ***	0.279 **	0.184 **	0.666 ***	0.650 ***	0.588 ***	0.152	0.240 **	-0.073	0.800 ***
N-W+	all	0.798 ***	0.530 ***	0.690 ***	0.696 ***	0.087	0.576 ***	0.114	0.734 ***	0.353 ***	0.046	0.814 ***	0.108	0.832 ***	0.854 ***	0.432 ***	-0.304 **	0.066	0.326 ***	0.111	0.266 **	0.621 ***	0.691 ***	0.414 ***	-0.127	0.184	-0.338 ***	0.803 ***
N+W-	all	-0.560 ***	0.127	-0.239 *	0.475 ***	0.884 ***	0.940 ***	0.486 ***	-0.512 ***	0.174	0.621 ***	0.302 **	0.719 ***	0.251 **	-0.172	0.700 ***	0.109	0.347 ***	-0.144	0.571 ***	0.364 ***	0.194 *	0.918 ***	0.649 ***	0.648 ***	0.778 ***	0.804 ***	0.887 ***
N-W-	all	0.233 *	0.750 ***	0.368 ***	0.592 ***	0.541 ***	0.749 ***	0.525 ***	0.162	0.102	0.261 **	0.389 ***	0.623 ***	0.337 ***	0.205 *	0.520 ***	-0.549 ***	-0.081	-0.211 *	0.241 *	-0.262 **	0.275 ***	0.745 ***	0.710 ***	0.388 ***	0.281 **	0.458 ***	0.672 ***
N+W+	#5	0.168	0.233	0.080	0.403	0.189	0.096	-0.296	0.448 *	0.239	0.026	0.192	0.029	0.495 *	0.173	-0.039	-0.026	-0.146	0.205	0.233	0.502 *	0.137	0.412	0.170	0.385	0.189	0.168	0.195
	#7	0.547 *	0.386	0.317	0.644 **	0.308	0.087	-0.114	0.526 *	0.525 *	0.298	0.535 *	0.433	0.614 **	0.543 *	0.024	-0.441	-0.057	-0.197	0.176	0.397	0.150	0.531 *	0.316	0.328	0.217	0.009	0.564 **
	#8	0.884 ***	0.158	0.666 **	0.714 ***	0.181	0.044	-0.487 *	0.798 ***	0.519 *	0.283	0.725 ***	0.202	0.908 ***	0.644 **	-0.117	-0.507 *	0.233	0.312	-0.035	0.704 ***	0.194	0.725 ***	0.525 *	0.439	0.435	0.017	0.830 ***
	#10	0.743 ***	0.692 ***	0.698 ***	0.761 ***	-0.349	0.314	-0.208	0.788 ***	0.648 **	0.454	0.777 ***	0.206	0.788 ***	0.579 **	-0.138	-0.680 ***	0.087	0.650 **	0.442	0.445 *	0.471 *	0.683 ***	0.753 ***	0.123	0.502 *	-0.010	0.811 ***
	#11	0.523 *	0.564 **	0.406	0.654 **	0.091	0.392	0.380	0.585 **	0.492 *	0.474 *	0.535 *	0.144	0.702 ***	0.454 *	-0.137	-0.039	0.466 *	0.176	0.415	0.308	0.424	0.535 *	0.662 **	0.075	0.272	-0.005	0.735 ***
	#13	0.117	0.301	-0.099	0.381	0.422	0.103	0.212	0.035	0.263	0.026	0.068	0.103	0.566 **	0.328	0.126	-0.050	-0.105	0.093	0.290	0.186	-0.017	0.169	0.375	-0.291	-0.434	-0.554 *	0.083
N-W+	#5	0.164	0.358	-0.139	0.358	-0.058	0.648 *	0.576	0.345	0.248	0.406	0.285	-0.355	0.406	0.527	0.152	-0.079	0.164	0.515	0.079	0.115	0.091	0.236	0.018	0.200	0.236	0.261	0.503
	#7	0.717 ***	-0.256	0.352	0.050	-0.484 *	0.254	-0.045	0.630 **	0.102	0.556 *	0.614 **	-0.344	0.755 ***	0.780 ***	0.541 *	0.098	-0.227	0.494 *	-0.224	0.707 ***	0.083	0.385	0.173	-0.066	0.439	-0.468 *	0.714 ***
	#8	0.678 **	0.005	0.645 **	0.361	-0.212	0.361	-0.612 **	0.707 ***	0.245	0.143	0.465 *	-0.297	0.632 **	0.665 **	0.445 *	0.405	0.101	0.261	0.191	0.677 **	0.576 **	0.531 *	0.278	0.089	0.681 ***	-0.114	0.588 **
	#10	0.698 ***	0.174	0.501 *	0.337	0.079	0.239	-0.215	0.585 **	0.472 *	-0.063	0.901 ***	-0.108	0.749 ***	0.800 ***	0.495 *	0.123	0.271	0.565 **	0.021	0.442	0.726 ***	0.522 *	0.086	0.063	0.224	-0.232	0.696 ***
	#11	0.850 ***	0.586 **	0.659 **	0.740 ***	-0.043	0.519 *	-0.176	0.827 ***	0.429	-0.122	0.928 ***	-0.118	0.914 ***	0.920 ***	0.450 *	-0.344	0.341	0.498 *	0.362	0.797 ***	0.874 ***	0.795 ***	0.689 ***	-0.168	0.323	0.214	0.893 ***
	#13	0.669 **	0.803 ***	0.460	0.665 **	0.200	0.671 **	0.890 ***	0.373	0.720 ***	0.702 ***	0.893 ***	0.713 ***	0.805 ***	0.872 ***	-0.717 ***	0.451 *	0.438	0.660 **	0.517 *	0.430	0.826 ***	0.859 ***	0.768 ***	-0.373	0.367	-0.383	0.911 ***
N+W-	#5	-0.018	0.588	-0.333	0.733 *	0.847 **	0.794 **	0.297	-0.467	0.479	-0.164	0.152	0.669 *	0.418	-0.455	0.964 ***	0.709 *	-0.285	-0.063	0.806 **	0.709 *	-0.345	0.891 ***	0.818 **	0.830 **	0.830 **	0.915 ***	0.418
	#7	-0.618 *	0.779 ***	-0.379	0.907 ***	0.804 ***	0.893 ***	-0.361	0.171	0.014	0.211	-0.082	0.771 ***	0.700 **	-0.400	0.425	-0.557 *	0.100	0.354	0.743 **	0.468	0.511	0.918 ***	0.900 ***	0.896 ***	0.882 ***	0.896 ***	0.725 **
	#8	-0.710 ***	0.816 ***	-0.533 *	0.945 ***	0.831 ***	0.932 ***	-0.159	-0.223	-0.553 *	0.877 ***	0.366	0.680 ***	0.737 ***	-0.450 *	0.924 ***	0.561 *	0.624 **	-0.240	0.424	-0.253	0.723 ***	0.914 ***	0.808 ***	0.773 ***	0.864 ***	0.893 ***	0.967 ***
	#10	-0.803 ***	-0.186	-0.737 ***	0.642 **	0.896 ***	0.955 ***	0.630 **	-0.678 **	-0.211	0.576 **	0.222	0.831 ***	-0.017	-0.427	0.765 ***	0.589 **	0.351	-0.518 *	0.418	0.287	0.449 *	0.887 ***	0.789 ***	0.812 ***	0.958 ***	0.899 ***	0.872 ***
	#11	-0.868 ***	-0.603 **	-0.639 **	0.003	0.920 ***	0.949 ***	0.180	-0.857 **	-0.475 *	0.223	-0.155	0.686 ***	-0.343	-0.582 **	0.386	0.517 *	0.179	-0.547 *	0.239	0.268	-0.032	0.777 ***	0.149	0.660 **	0.877 ***	0.908 ***	0.829 ***
	#13	-0.755 ***	-0.854 ***	-0.565 **	-0.743 ***	0.589 **	0.833 ***	0.129	-0.785 ***	0.033	0.496 *	-0.594 **	0.000	0.126	-0.615 **	0.829 ***	-0.077	0.475 *	-0.265	0.504 *	0.735 ***	-0.433	0.795 ***	0.215	0.603 **	0.770 ***	0.890 ***	0.839 ***
N-W-	#5	-0.358	0.091	-0.576	0.055	-0.174	0.248	-0.127	-0.382	-0.018	0.042	0.042	0.061	0.055	-0.139	0.200	0.321	-0.273	0.333	0.030	-0.152	-0.006	0.200	0.261	0.248	0.430	0.527	0.006
	#7	-0.417	0.795 ***	0.053	0.814 ***	0.695 ***	0.583 **	-0.345	-0.438	0.380	-0.008	0.380	0.827 ***	0.457 *	-0.540 *	0.732 ***	-0.471 *	0.155	0.002	0.741 ***	-0.257	0.729 ***	0.755 ***	0.771 ***	0.768 ***	0.794 ***	0.662 **	-0.114
	#8	-0.367	0.746 ***	0.158	0.704 ***	0.664 **	0.514 *	-0.456 *	-0.188	0.093	0.042	0.120	0.691 ***	0.284	-0.299	0.653 **	-0.335	0.371	-0.148	0.693 ***	-0.247	0.627 **	0.644 **	0.770 ***	0.789 ***	0.725 ***	0.645 **	-0.035
	#10	-0.513 *	0.701 ***	-0.471 *	0.353	0.428	0.400	0.450 *	-0.489 *	-0.358	0.174	-0.271	0.659 **	-0.463 *	-0.456 *	0.227	-0.036	0.174	-0.542 *	0.128	-0.546 *	-0.242	0.568 **	0.720 ***	0.707 ***	0.474 *	0.716 ***	0.650 **
	#11	-0.408	0.597 **	-0.430	-0.093	0.367	0.656 **	0.782 ***	-0.484 *	-0.377	0.364	-0.373	0.667 **	-0.427	-0.493 *	0.186	0.250	0.024	-0.083	-0.017	-0.505 *	-0.247	0.414	0.644 **	0.719 ***	0.454 *	0.755 ***	0.666 **
	#13	-0.329	-0.227	-0.603 **	-0.352	0.898 ***	0.573 **	-0.505 *	-0.511 *	-0.241	0.205	-0.624 **	-0.344	-0.296	-0.561 *	0.722 ***	-0.476 *	-0.352	-0.223	0.409	0.047	-0.720 ***	0.424	0.770 ***	0.767 ***	0.502 *	0.827 ***	0.380

Results are presented for leaf ranks #5, #7, #9, #10, #11 and #13 during 14 days of treatment.

Spearman coefficients: *, $P \leq 0.05$; **, $P \leq 0.01$; ***, $P \leq 0.001$

Abbreviations. Glu: glutamate; Gln: glutamine; Asp: aspartate; Asn: asparagine; HO-Pro: hydroxyproline; His: histidine; SMCSO: S-methylcysteine sulfoxide; Ser: serine; Arg: arginine; Gly: glycine; Hser: homoserine; β-Ala: β-alanine; Thr: threonine; α-Ala: α-alanine; GABA: γ-aminobutyric acid; SMC: S-methylcysteine; Orn: ornithine; Cys: cystine; Lys: lysine; Tyr: tyrosine; Met: methionine; Val: valine; Ile: isoleucine; Leu: leucine; Phe: phenylalanine; Trp: tryptophan; ΣAAs : total free amino acids
